# Serum metabolic profiling implicates mTOR activation and insulin resistance in the development of pulmonary hypertension in a rat model of pressure overload-induced heart failure

**DOI:** 10.1016/j.gendis.2023.101097

**Published:** 2023-09-14

**Authors:** Qi Zhou, Chao Tang, Xinke Xu, Junhao Hu, Shengsen Yao, Bo Dong, Xiaojing Wu

**Affiliations:** a Department of Cardiology of the Fifth Affiliated Hospital of Southern Medical University, Guangzhou, Guangdong 510999, China; b Cardiovascular Department of Shenzhen University General Hospital, Shenzhen, Guangdong 518055, China; c Cardiovascular Department of Chongqing Medical University Second Affiliated Hospital, Chongqing 400010, China

Pulmonary hypertension (PH) due to left heart failure (HF) accounts for approximately half of PH cases worldwide. It has been demonstrated that PH is a frequent complication of HF but is difficult to recognize in its early stage because it is characterized by nonspecific symptoms including shortness of breath and decreased tolerance of activity. Moreover, nearly all multicentric clinical trials targeting pulmonary circulation in PH-HF have reported negative results.^1^ Thus, there are no effective methods for diagnosing early PH, and the current treatment of PH-HF is insufficient.

HF is the most common cause of PH. PH's development is triggered by the direct retrograde transmission of the left atrial pressure to the pulmonary circulation in the initial stage. Furthermore, during the development of left heart dysfunction, many cytokines, vasoactive substances, and metabolites contribute to cardiac remodeling. Metabolic changes have been reported to appear before the structural changes of cardiac remodeling, and metabolic dysregulation is considered a contributing factor to HF's development. However, the metabolites that correlate with pulmonary hemodynamic changes in patients with HF remain largely unknown. To further elucidate the metabolic mechanisms of PH due to HF (PH–HF), we used a left heart overloaded rat model induced by transverse aortic constriction (TAC) surgery and observed the correlations of metabolic patterns and metabolites with pulmonary hemodynamic changes during the progression of left ventricular dysfunction.

Forty male Sprague–Dawley rats were randomly selected to undergo TAC or sham surgery. Finally, 18 animals in the sham group and 19 animals in the TAC group survived and were used for the experiment. The rats were subjected to further examinations immediately (0 W, *n* = 6), 3 weeks (3 W, *n* = 6), and 9 weeks (9 W, *n* = 7) after surgery. At 3 weeks after TAC surgery, the rats exhibited high blood pressure, a normal left ventricular ejection fraction (LVEF), and normal mean pulmonary arterial pressure (mPAP) compared with the 0 W group. The thickness of the left ventricular systolic posterior wall (LVPW) and intraventricular septum (IVS) tended to be elevated but were not significantly different from the rats at 0 W (Table S1). However, the cross-sectional area of cardiomyocytes increased in the histological assessment, suggesting that there was subclinical compensated cardiomyocyte hypertrophy (Fig. 1A, B). The rats developed significant increases in the thickness of the LVPW and IVS, a decrease in the LVEF, and an increase in the mPAP at 9 weeks compared with 3 weeks after TAC surgery (Table S1). Histological assessments demonstrated thickened walls of the small pulmonary arteries in the rats at 9 weeks after TAC surgery (Fig. 1A, C). These data indicated that the rats had developed pronounced PH-HF at 9 weeks after TAC surgery.

**Figure 1:**
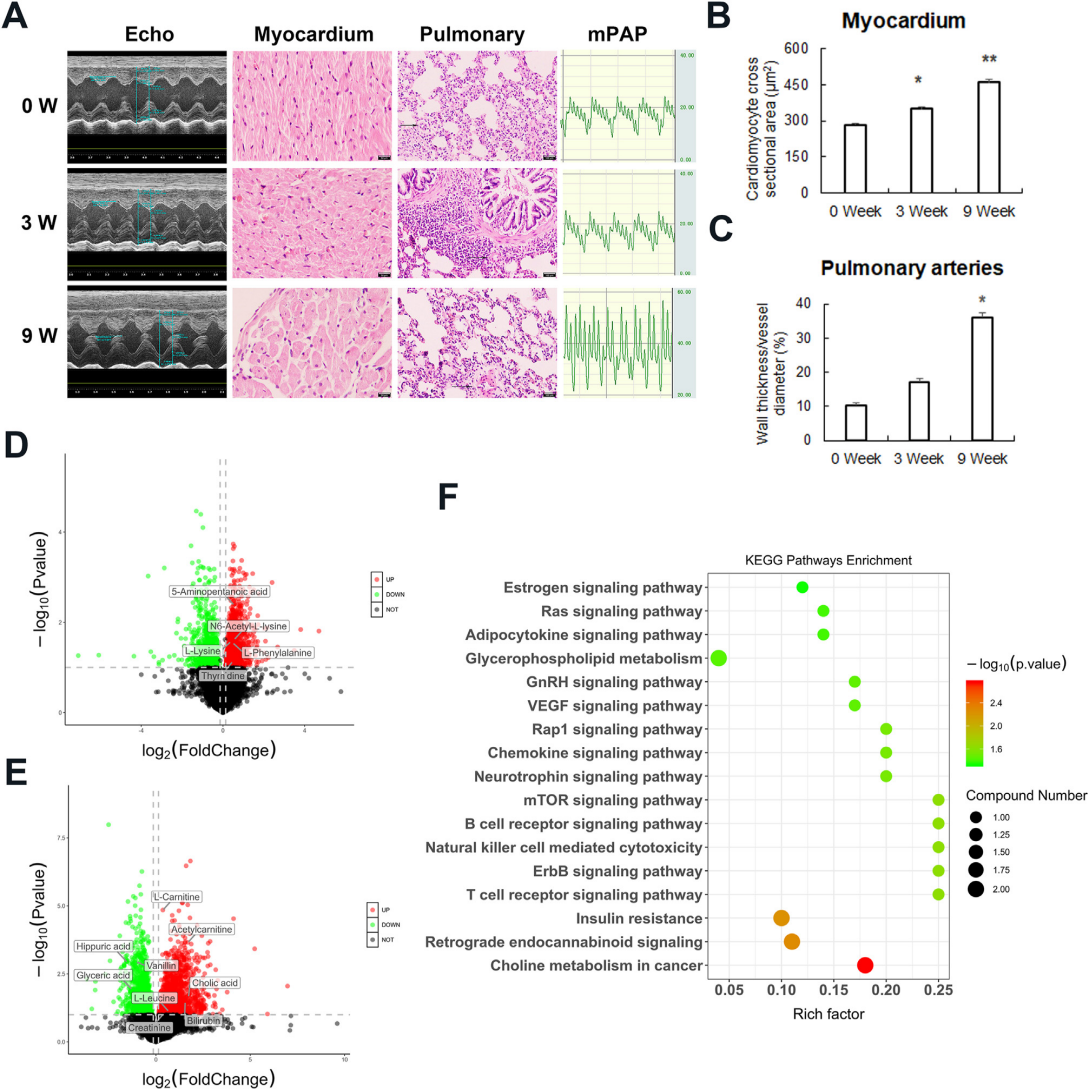
Metabolic changes associated with the formation of pulmonary hypertension in pressure overload-induced heart failure. **(A)** Representative images of echocardiograms, hematoxylin/eosin (HE) staining of the myocardium and small pulmonary arteries, and mean pulmonary artery pressure (mPAP) assayed using a right cardiac catheter at 0, 3, and 9 weeks after TAC surgery. The bar was 100 μm for HE staining of the myocardium; the bar was 50 μm for HE staining of the pulmonary tissue. **(B)** The cross-sectional area of cardiomyocytes. **(C)** Wall thickness/vessel diameter of small pulmonary arteries. **(D, E)** The clustering results of a hierarchical cluster analysis based on the significantly different metabolites. (D) The cluster results of 0 *vs*. 3 weeks post-TAC. (E) The cluster results of the 3 weeks post-TAC findings *vs*. the 9 weeks post-TAC findings. **(F)** Comparison of the enriched KEGG pathways in the 3 W and 9 W post-TAC findings presented as a bubble diagram based on significantly different metabolites. The data were expressed as mean ± standard error. *n* = 6 for the 0 W and 3 W groups; *n* = 7 for the 9 W group. ^∗^*P* < 0.05, ^∗∗^*P* < 0.01.

We then analyzed the metabolomic pattern and metabolites present by an untargeted metabolomics analysis. The plasma metabolites were detected by ultra-performance liquid chromatography with quadrupole time-of-flight mass spectrometry. An orthogonal partial least squares discriminant analysis was conducted to investigate the association of the metabolite patterns with PH's development. A clear separation among the groups at 0 W, 3 W, and 9 W was observed, and the different metabolomic patterns could be used to separate the plasma samples into normal, compensated left ventricular hypertrophy, and PH-HF groups. Significantly different metabolites were obtained based on the orthogonal partial least squares discriminant analysis, with the values of the variable importance in the projection > 1 and *P* < 0.05 among groups at different time points used to indicate significance. The findings are shown by volcano plots (Fig. 1D, E).

Thirteen metabolites, including amino acids, pyrimidine, choline, and glycerophospholipids, were changed in the 3 W group compared with the 0 W group. The significantly different metabolites included 5-aminopentanoic acid, l-lysine, N6-acetyl-l-lysine, and l-phenylalanine. There were 25 differentially abundant metabolites at 9 W compared with 3 W in the PH-HF group, with no significant differences observed for the different time points in the sham group. The significantly different metabolites included l-carnitine, acetylcarnitine, cholic acid, bilirubin, creatinine, hippuric acid, glyceric acid, l-leucine, and vanillin (Table S2). These data suggest that there are measurable metabolic changes that occur during the formation of PH-HF.

A KEGG enrichment analysis was subsequently performed to reveal the mechanism(s) underlying PH's development during the progression of left heart dysfunction. Lysine degradation was the most disturbed metabolic pathway contributing to cardiac hypertrophy at 3 W after TAC surgery. More pathways, including insulin resistance and mTOR, were disturbed at 9 W after TAC surgery compared with 3 W after surgery (Fig. 1F). Despite the well-acknowledged correlation between insulin resistance and coronary artery disease, evidence linking pulmonary vascular diseases with insulin signaling was only recently reported.^2^ The important regulatory role of mTOR in both cardiac hypertrophy and pulmonary vascular remodeling has also been recognized. Our study showed that both insulin resistance and mTOR were activated during the transition from compensated cardiomyocyte hypertrophy to the formation of PH.

Increased activity of the sympathetic system is an important feature contributing to the progression of HF, and the activation of β-adrenergic receptor signaling might lead to the development of insulin resistance.^3^ In our study, however, the only metabolite identified related to insulin resistance was acetylcarnitine. Based on our initial findings, the plasma level of acetylcarnitine was further assayed by enzyme-linked immunosorbent assays. Consistent with the metabolomic data, the levels of acetylcarnitine were significantly increased at 9 weeks compared with these at 3 weeks after surgery (12.04 ± 1.42 μg/mL *vs*. 4.14 ± 0.43 μg/mL, *P* < 0.01).

Abnormal free fatty acid metabolism has also been reported to be involved in insulin resistance. Proinflammatory cytokines impair the suppression of adipose tissue lipolysis, leading to the release of free fatty acid into the circulation. Incomplete fatty acid *β*-oxidation and the subsequent increase in acylcarnitine species might be linked to insulin resistance.^4^ Our present study thus revealed a new mechanism by which the progression of HF may lead to insulin resistance, in addition to β-adrenergic receptor signaling. Acetylcarnitine is an acetic acid ester of carnitine that facilitates the movement of acetyl CoA into the mitochondria during fatty acid oxidation. Therefore, the elevation of acetylcarnitine might not only participate in the development of PH but also serve as a biomarker for PH-HF.

Branched-chain amino acids are regulators of mTOR activation. In our study, l-leucine was related to mTOR activation, and its relative level increased 1.6-fold with a value of the variable importance in the projection of 4.08 at 9 weeks when compared with the findings 3 weeks after TAC surgery (Table S2). Leucine has already been reported to be the most potent activator for insulin resistance.^5^ Moreover, insulin and leucine have an additive effect on mTOR phosphorylation. l-leucine might mediate the crosstalk between insulin resistance and mTOR activation.

Metabolites are considered useful tools in the early diagnosis and treatment of diseases because they can be easily and noninvasively detected. Metabolomic analyses of multiple cardiovascular diseases have identified target metabolites that could provide insights into the mechanisms of disease progression and potential targets for prevention or treatment. Our present studies showed that during the progression of left heart dysfunction, changes in metabolites reflect changes in pulmonary hemodynamic parameters and may also be involved in the development of PH-HF.

In summary, by using a rat model of TAC, we showed that the metabolic pattern and metabolites were significantly different between compensated cardiac hypertrophy and PH-HF. As demonstrated by the changes that occurred during the transition from compensated cardiomyocyte hypertrophy to PH-HF, dysfunctional insulin resistance and mTOR activation might participate in the development and progression of PH. Our study provides a novel method for noninvasively distinguishing PH from HF based on metabolic patterns and metabolite assays. In addition, the metabolites identified in this study might represent potential targets for the prevention or treatment of PH-HF.

## Conflict of interests

The authors declare no conflict of interests.

## Funding

This study was supported by grants from the Science, Technology, and Innovation Commission of Shenzhen Municipality, Guangdong, China (No. JCYJ20190808122207499), the Natural Science Foundation of Guangdong Province, China (No. 2019A1515011421), and the National Natural Science Foundation of China (No. NSFC 81270109) to X Wu. The funders had no role in the study design, in the collection, analysis, and interpretation of data, in the writing of the manuscript, and in the decision to submit the article for publication.
