# Supplementary material for: Serum metabolic profiling implicates mTOR activation and insulin resistance in the development of pulmonary hypertension in a rat model of pressure overload-induced heart failure
Source: Genes Dis. 2023 Sep 14;11(5):101097. doi: 10.1016/j.gendis.2023.101097 (PMC11176641; doi:10.1016/j.gendis.2023.101097)
Supplement: Multimedia component 1 [file mmc1.docx]

**Table S1. Echocardiographic and pulmonary hemodynamic changes of rats subjected to TAC surgery**

|  | **0 W**  **(n=6)** | **3 W**  **(n=6)** | **9 W**  **(n=7)** |
| --- | --- | --- | --- |
| BW (g) | 258±18 | 274±15 | 429±13 |
| HR (BPM) | 385±8 | 407±3 | 391±5 |
| BP (mmHg) | 132±5.85 | 158±16.37** | 201±19.35** |
| IVS;d(mm) | 1.45±0.11 | 1.71±0.09 | 2.86±0.34* |
| IVS;s(mm)  LVID;d(mm) | 2.51±0.16  5.42±0.38 | 2.86±0.22  5.94±0.29 | 4.43±0.29*  6.47±0.88* |
| LVID;s(mm) | 2.69±0.52 | 2.85±0.36 | 3.56±0.62* |
| LVPW;d(mm) | 1.44±0.27 | 1.95±0.22 | 2.64±0.30* |
| LVPW;s(mm) | 2.65±0.17 | 3.04±0.28 | 3.71±0.54* |
| LVEDV(ul) | 164.27±25.78 | 198.61±24.96 | 278.57±43.09* |
| LVEF(%) | 82.91±5.79 | 86.67±4.39 | 67.09±2.61* |
| LVFS(%) | 54.81±8.25 | 61.09±7.32 | 42.67±4.48* |
| LV Mass(mg) | 517.38±35.26 | 652.28±30.52 | 1224.96±166.02* |
| mPAP(mmHg) | 14.15±0.13 | 15.81±0.78 | 30.08±1.07* |
| RVSP | 21.70±1.06 | 23.73±0.97 | 42.65±0.87* |

Data presented as Mean±SE for 0W,3W and 9W group.

BW：Body weight；HR：Heart rate；BP：blood pressure; IVS;d: the diastolic thickness of intraventricular septum; IVS;s: the systolic thickness of intraventricular septum; LVID;d: left end diastolic diameter; LVID;s: left end systolic diameter; LVPW;d: left ventricular diastolic posterior wall thickness; LVPW;s: left ventricular systolic posterior wall thickness; :LVFS: percent left ventricular fractional shortening; LVEDV： left ventricular end diastolic volume; LVEF: left ventricular ejection fraction; LV Mass: left ventricular mass; mPAP: mean pulmonary artery pressure; RVSP: right ventricular systolic pressure。

*P<0.05 vs 0 W, **P<0.01 vs 0W

**Table S2. Identified significant different metabolites between 3w and 9w**

| **Name of metabolites** | **Category** | **VIP** | **FC** | **p** |
| --- | --- | --- | --- | --- |
| L-Carnitine | Quaternary ammonium salts | 9.49 | 1.29 | <0.001 |
| Acetylcarnitine | Fatty acid esters | 14.65 | 3.24 | <0.001 |
| L-Palmitoylcarnitine | Fatty acid esters | 4.17 | 2.86 | <0.001 |
| 1,2-dioleoyl-sn-glycero-3-phosphatidylcholine | others | 8.82 | 0.49 | 0.007 |
| (3-Carboxypropyl)trimethylammonium cation | others | 2.56 | 1.55 | 0.010 |
| Dihomo-gamma-linolenoyl-EA | others | 2.10 | 4.25 | 0.015 |
| Uracil | Pyrimidines | 1.13 | 0.40 | 0.015 |
| Triethanolamine | Alkanolamines | 1.64 | 4.14 | 0.019 |
| SOPC | Glycerophosphocholines | 1.06 | 1.51 | 0.028 |
| 1-Palmitoylglycerol | others | 1.67 | 1.24 | 0.029 |
| Bilirubin | Bilirubins | 1.04 | 2.83 | 0.036 |
| epsilon-Caprolactam | others | 2.48 | 0.73 | 0.043 |
| Confertifoline | others | 4.88 | 0.59 | <0.001 |
| 2-Methylbenzoic acid | others | 1.21 | 0.68 | <0.001 |
| D(-)-beta-hydroxy butyric acid | others | 4.26 | 4.20 | 0.001 |
| Hippuric acid | Benzoic acids | 3.80 | 0.35 | 0.001 |
| Vanillin | Methoxyphenols | 2.81 | 0.49 | 0.001 |
| Pentobarbital | others | 14.21 | 0.56 | 0.009 |
| Glyceric acid | Carbohydrates and carbohydrate conjugates | 2.00 | 0.41 | 0.011 |
| 3-Methoxy-4-Hydroxyphenylglycol Sulfate | Methoxyphenols | 2.17 | 1.82 | 0.020 |
| Cholic acid | Bile acids | 5.08 | 3.22 | 0.021 |
| 1,4-Dihydroxybenzene | Benzenediols | 1.69 | 0.34 | 0.023 |
| D-Threitol | Carbohydrates | 1.32 | 1.53 | 0.027 |
| Dihydrothymine | Pyrimidines | 1.93 | 0.59 | 0.047 |
| L-Leucine | Amino acids | 4.08 | 1.60 | 0.045 |

SOPC：1-Stearoyl-2-oleoyl-sn-glycerol 3-phosphocholine
